# Esophageal Squamous Cell Papilloma: A Case Report of Controversial Rare Benign Esophageal Lesion

**DOI:** 10.34172/mejdd.2025.419

**Published:** 2025-04-30

**Authors:** Caroline Tanadi, Aileen Alessandra Suryohusodo, Kevin Tandarto, Maria Gabrielle Simadibrata, Randy Adiwinata, Paulus Simadibrata, Marcellus Simadibrata

**Affiliations:** ^1^School of Medicine and Health Sciences, Atma Jaya Catholic University of Indonesia, Jakarta, Indonesia; ^2^Faculty of Medicine, Universitas Indonesia, Jakarta, Indonesia; ^3^Gastrointestinal Cancer Center, MRCCC Siloam Semanggi Hospital, Jakarta, Indonesia; ^4^Department of Internal Medicine, Abdi Waluyo Hospital, Jakarta, Indonesia; ^5^Division of Gastroenterology, Department of Internal Medicine, Faculty of Medicine, Universitas Indonesia, Cipto Mangunkusumo Hospital, Jakarta, Indonesia

**Keywords:** Papilloma, Squamous cell, Esophagus, Endoscopy

## Abstract

Esophageal squamous cell papilloma (ESP) is a benign tumor that is rarely found in the esophagus. Human papillomavirus (HPV) has been known to play a role in the development of ESP. In most cases, ESP is asymptomatic but may also manifest with dysphagia, heartburn, and epigastric pain. Although rare, ESP has the potential to become malignant; thus, early detection and removal of the tumor is essential. Here, we report a rare case of ESP in a 29-year-old woman.

## Introduction

 Esophageal squamous cell papilloma (ESP) is a controversial, rare, benign growth of the esophagus. The prevalence for ESP reaches up to 0.45%, with its peak prevalence found in the age groups 2-17 years and 40-50 years.^1^ Although the etiology of ESP is still unclear, some studies have linked ESP with human papillomavirus (HPV), chronic inflammation, and genetics.^2,3^ Notably, ESP has been reported to have the potential of progressing into malignancy, especially squamous cell carcinoma (SCC).^1^ Although there have been a few reports on ESP, most of them presented classical ESPs that are solitary and located primarily at the mid-distal esophagus. Furthermore, most of these cases were either asymptomatic and discovered incidentally or complained of dysphagia, a typical manifestation of ESP.^1,4-7^ Conversely, we report an unusual case of multiple ESPs, both found in the upper esophagus, in a 29-year-old woman who had a 2-year history of heartburn in Indonesia. Additionally, this report highlights the importance of esophagogastroduodenoscopy in chronic heartburn cases to exclude other potential diagnoses, such as ESP, which could potentially progress into malignancy, if left untreated.

## Case Report

**Figure 1:**
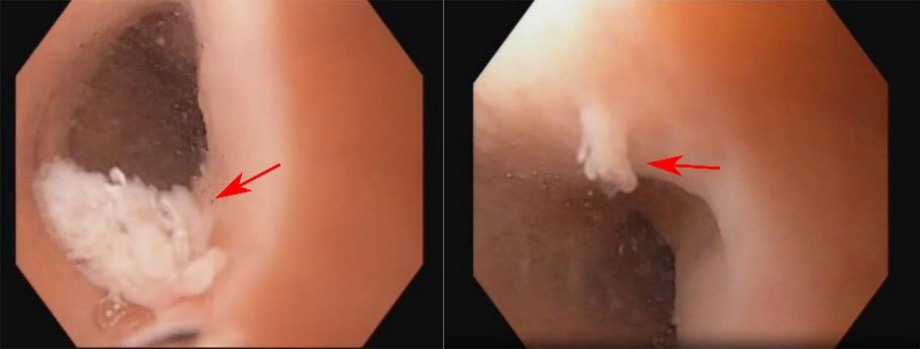
Two esophageal squamous cell papillomas appearing as well-demarcated, wart-like sessile nodules on esophagogastroduodenoscopy

**Figure 2:**
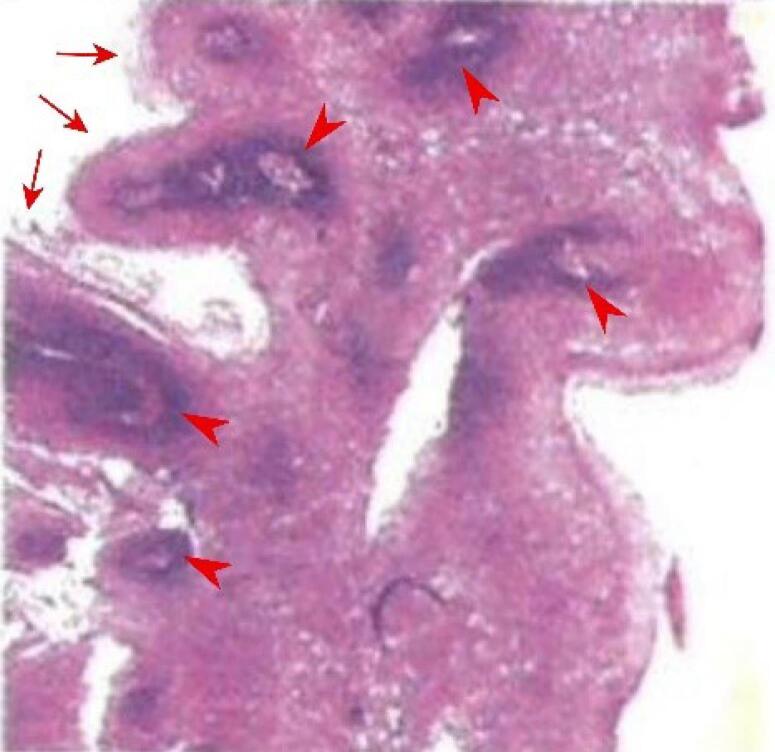
Papillomatous tissue with acanthotic squamous epithelium (arrows), koilocytes, and lymphocyte infiltrates in the stroma (arrowheads) on histopathological examination with H&E stains at 40x magnification

 A 29-year-old woman presented with a 2-year history of heartburn. She denied difficulty swallowing or weight loss. After receiving the patient’s informed consent, we performed an esophagogastroduodenoscopy, revealing two well-demarcated, wart-like-sessile nodules measuring 3-4 mm in the upper thoracic esophagus (Figure 1); and antral gastritis. Esophageal biopsies of lesions were performed via cold-biopsy-forceps, and the lesions disappeared immediately. Histopathological examination revealed papillomatous tissue with acanthotic squamous epithelium, koilocytes ( + ), lymphocytes in the stroma, and no signs of malignancy consistent with ESP (Figure 2).

## Discussion

 ESP is a relatively rare benign esophageal tumor with a prevalence that varies from 0.01% to 0.45%. It can be found in two age groups, namely, 2 to 17 years and 40 to 50 years. This condition seemed to affect both men and women equally. In our case, we found the ESP in a woman outside of both age groups. Although most cases of ESP are asymptomatic, when large enough, it may result in dysphagia. Some patients may also complain of heartburn, epigastric pain, and weight loss. It is usually discovered incidentally during an esophagogastroduodenoscopy and is commonly located in the distal part of the esophagus. The classical features of ESP were surface vessel crossing, wart-like projections, and exophytic growth.^1,2^ Our patient had recurrent heartburn and showed classical ESP features.

 Although the exact cause of ESP is still unknown, HPV infection, chronic mucosal irritation, and genetics have been proposed as its probable etiology.^2,3^ The most common HPV genotypes associated with ESP were HPV6 and HPV11. Although HPV infection has been proposed to be the cause of ESP, it is still controversial since only 11-57% of ESP tested positive for HPV.^3^ A meta-analysis also found that only 30.9% of ESP were HPV-positive.^8^ Thus, HPV may not be the only factor that causes ESP. Chronic mucosal irritation due to chemical or mechanical causes has also been associated with ESP. One common chemical cause of mucosal irritation is gastroesophageal reflux disease (GERD). Animal studies have shown that esophageal or duodenal reflux appeared to induce ESP. This may explain why the most common site for ESP is at the distal part of the esophagus. Other than GERD, mechanical injuries such as those caused by metal stents, nasogastric intubation, and esophageal dilatation may also contribute to the development of ESP. Genetics may also play a role in the pathogenesis of ESP. Goltz syndrome, characterized by mutations in the PORCN gene, and angioma serpiginosum, a congenital nevoid disorder that can be autosomal or X-linked dominant, have been associated with ESP.^2,3^

 ESP is commonly solitary and found at the mid-distal esophagus with a size < 5 mm.^9^ However, our case demonstrated two lesions in the upper esophagus. This finding aligned with the results of several studies whereby a minority of patients may present with multiple ESPs, although the number of lesions is usually less than five.^10,11^ ESP can be identified by narrow-band imaging endoscopy with a positive predictive value of 88.2%. It is characterized by the triad of exophytic growth, wart-like projections, and surface vessel crossing.^12^ The histological examination typically reveals a fibrovascular core extending from the lamina propria, forming finger-like projections without penetrating the submucosa with an exophytic pattern. These projections are surrounded by hyperplastic squamous epithelium, sometimes with koilocytosis (squamous cells with perinuclear halo, cytoplasmic thickening, and irregular nuclei). The overlying squamous epithelium generally has an orderly maturation, resembling a normal esophagus. In contrast, histologic features of squamous dysplasia include nuclear atypia in squamous cells, loss of normal cell polarity, and abnormal tissue maturation, with no invasion through the basement membrane. SCC is characterized by uncontrolled growth of neoplastic squamous epithelial cells that penetrate the basement membrane, extending into or beyond the lamina propria.^9^

 The malignancy potential of ESP is still controversial. While generally being thought of as a benign condition, some studies associate ESP with dysplasia and SCC. Cho and colleagues reported a case of ESP progression to cancer after 2 years.^5^ Several cohort studies also demonstrated the malignant development of ESP. Approximately 1.3-1.5% of ESP cases progressed into SCC after 2 years of follow-up.^10,13^ De Cristofaro and others also demonstrated a case of carcinoma in situ arising from a papillomatous lesion.^14^ On the other hand, a cohort study by Carr and co-workers showed no esophageal malignancy after a 2-year follow-up in 25 ESP cases.^15^ Similarly, Jideh and colleagues also demonstrated that malignant cases in the cohort were not preceded by papilloma.^16^

 As stated above, ESP can potentially progress into SCC. This proves that although it is a rare and benign tumor with small risks of progressing into a malignant tumor, performing early detection and eradication is still extremely vital. Thus, the total removal of ESP and endoscopic surveillance are warranted.^17^ We removed the ESP using cold-biopsy-forceps and no dysplasia was found on histopathological examination.

 Several medicine methods have been proposed and reported for ESP management with variable effectivity, such as interferon alpha administration and (S)-l-(3-hydroxy-2-phosphonylmethoxypropy1) cytosine injection. ^18^ Endoscopic therapies may also be employed in treating ESP. These therapies include argon plasma coagulation, Nd-YAG laser, radiofrequency ablation, cryotherapy, and photodynamic therapy. The coagulation, laser, and ablation will induce rapid coagulative necrosis in ESP. Similarly, cryotherapy treats ESP by applying liquid nitrogen, which causes tissue deterioration and necrosis. Other viable options include endoscopic mucosal resection and endoscopic submucosal dissection. Given the risk of malignancy progression, ESP with severe dysplasia or confirmed esophageal SCC should be treated more aggressively, such as by esophagectomy.^2^

 Although various methods exist to treat ESP, it can recur at a rate of 0.65%-3.8%. Moreover, surveillance may be required in certain cases due to its malignant potential. Although there has not been a clear guideline on ESP surveillance, studies have suggested that patients with ESP greater than 0.5 cm with atypia or dysplasia should be monitored closely via endoscopy every 3 months.^10,19^

 In conclusion, we hereby present a rare case of multiple ESPs found in the upper esophagus in a 29-year-old woman presenting with chronic heartburn. Although it is usually a benign lesion, ESP does have the potential to develop into SCC. Thus, early detection, eradication of the lesion, and surveillance should be conducted.
